# Supplementary material for: CTCF depletion decouples enhancer-mediated gene activation from chromatin hub formation
Source: Nat Struct Mol Biol. 2025 May 13;32(7):1268–81. doi: 10.1038/s41594-025-01555-z (PMC12263429; doi:10.1038/s41594-025-01555-z)
Supplement: Supplementary file 1 — Reporting Summary [file 41594_2025_1555_MOESM1_ESM.pdf]

nature portfolio

Corresponding author(s): A. Marieke Oudelaar

Last updated by author(s): Apr 4, 2025

Reporting Summary

Nature Portfolio wishes to improve the reproducibility of the work that we publish. This form provides structure for consistency and transparency in reporting. For further information on Nature Portfolio policies, see our Editorial Policies and the Editorial Policy Checklist.

Statistics

For all statistical analyses, confirm that the following items are present in the figure legend, table legend, main text, or Methods section.

n/a	Confirmed	
☐	☒	The exact sample size (*n*) for each experimental group/condition, given as a discrete number and unit of measurement
☐	☒	A statement on whether measurements were taken from distinct samples or whether the same sample was measured repeatedly
☐	☒	The statistical test(s) used AND whether they are one- or two-sided<br>*Only common tests should be described solely by name; describe more complex techniques in the Methods section.*
☒	☐	A description of all covariates tested
☒	☐	A description of any assumptions or corrections, such as tests of normality and adjustment for multiple comparisons
☐	☒	A full description of the statistical parameters including central tendency (e.g. means) or other basic estimates (e.g. regression coefficient) AND variation (e.g. standard deviation) or associated estimates of uncertainty (e.g. confidence intervals)
☐	☒	For null hypothesis testing, the test statistic (e.g. *F*, *t*, *r*) with confidence intervals, effect sizes, degrees of freedom and *P* value noted<br>*Give P values as exact values whenever suitable.*
☒	☐	For Bayesian analysis, information on the choice of priors and Markov chain Monte Carlo settings
☒	☐	For hierarchical and complex designs, identification of the appropriate level for tests and full reporting of outcomes
☐	☒	Estimates of effect sizes (e.g. Cohen's *d*, Pearson's *r*), indicating how they were calculated

Our web collection on statistics for biologists contains articles on many of the points above.

Software and code

Policy information about availability of computer code

Data collection	Illumina NextSeq 550.
Data analysis	CapCruncher pipeline v.0.2.3 (https://github.com/sims-lab/CapCruncher); MCC pipeline v.1 (https://github.com/jojdavies/Micro-Capture-C), based on scripts available for academic use through the Oxford University Innovation software store (v1; https://process.innovation.ox.ac.uk/software/p/165294/micro-capture-c-academic/1); Bowtie2 v.2.3.5; HiC-Pro v.2.11.1; oligo design tool v.0.1.1b (https://oligo.readthedocs.io/en/latest/); MACS2 v.2.1.2; deepTools v.3.3.0; DESeq2 v.1.36.0; NGseqBasic pipeline v.1 (https://github.com/Hughes-Genome-Group/NGseqBasic/releases); R v.4.2.0; rtracklayer v.1.56.1; UCSC bigWigMerge v.2; nf-core/rnaseq pipeline v.3.12.0 (https://nf-co.re/rnaseq/3.12.0/); STAR v.2.6.1d; featureCounts v.2.0.6; cooltools v.0.5.4; FlowJo v.10.8.1.

For manuscripts utilizing custom algorithms or software that are central to the research but not yet described in published literature, software must be made available to editors and reviewers. We strongly encourage code deposition in a community repository (e.g. GitHub). See the Nature Portfolio guidelines for submitting code & software for further information.

Data

Policy information about availability of data

All manuscripts must include a data availability statement. This statement should provide the following information, where applicable:

- Accession codes, unique identifiers, or web links for publicly available datasets
- A description of any restrictions on data availability
- For clinical datasets or third party data, please ensure that the statement adheres to our policy

The Micro-Capture-C, Capture-C, Tri-C, ChIPmentation and RNA-seq datasets generated and analyzed for the current study are available from the Gene Expression Omnibus (GEO) as a SuperSeries under accession number GSE263641 (reviewer token: wxmfyuqqhrcptsh).
Reference genome for data from BLaER1 and BLaER1-CTCF-mAID cell lines was human assembly Dec. 2013 (GRCh38/hg38). Reference genome for Dosophila spike-in was D. melanogaster assembly Aug. 2014 (BDGP Release 6 + ISO1 MT/dm6). Index files were downloaded from Bowtie2 website (https://bowtie-bio.sourceforge.net/bowtie2/manual.shtml).

Research involving human participants, their data, or biological material

Policy information about studies with human participants or human data. See also policy information about sex, gender (identity/presentation), and sexual orientation and race, ethnicity and racism.

Reporting on sex and gender	n/a
Reporting on race, ethnicity, or other socially relevant groupings	n/a
Population characteristics	n/a
Recruitment	n/a
Ethics oversight	n/a

Note that full information on the approval of the study protocol must also be provided in the manuscript.

Field-specific reporting

Please select the one below that is the best fit for your research. If you are not sure, read the appropriate sections before making your selection.

☒ Life sciences ☐ Behavioural & social sciences ☐ Ecological, evolutionary & environmental sciences

For a reference copy of the document with all sections, see nature.com/documents/nr-reporting-summary-flat.pdf

Life sciences study design

All studies must disclose on these points even when the disclosure is negative.

Sample size	The data presented in the manuscript represent the averages of three biological replicates, which is standard in the field. No statistical method was used to pre-determine sample sizes. These sample sizes were chosen to generate data at sufficient depth and assess differences between conditions robustly. These sample sizes are sufficient, since the observed biological effects of interest are clearly detectable between conditions and robust across replicates. For Micro-Capture-C and Tri-C experiments, multiple technical replicates for each biological replicate were included to boost the complexity of the data.
Data exclusions	Two viewpoints (IFNGR2 and TASL) were excluded from the quantifications of Micro-Capture-C and Tri-C experiments due to poor data quality. The capture coordinates targeting promoter in Micro-Capture-C were: IFNGR2 - chr21:33403238-33403358, TASL - chrX:30577754-30577874 and in Tri-C targeting enhancer were: IFNGR2 - chr21:33216352-33216472, TASL - chrX:30596991-30597111.
Replication	All experiments based on sequencing data were performed for n=3 biologically independent samples as described and all attempts were successful.
Randomization	Samples were randomly allocated into different experimental groups prior to their treatment with auxin or control conditions.
Blinding	All samples were analyzed with the same pipelines, in which results are generated by scripts without interference of the researchers. Since potential expectations of the researchers cannot influence the data analysis and results, blinding is not relevant to this study.

Reporting for specific materials, systems and methods

We require information from authors about some types of materials, experimental systems and methods used in many studies. Here, indicate whether each material, system or method listed is relevant to your study. If you are not sure if a list item applies to your research, read the appropriate section before selecting a response.

Materials & experimental systems

n/a	Involved in the study
☐	☒ Antibodies
☐	☒ Eukaryotic cell lines
☒	☐ Palaeontology and archaeology
☒	☐ Animals and other organisms
☒	☐ Clinical data
☒	☐ Dual use research of concern
☒	☐ Plants

Methods

n/a	Involved in the study
☐	☒ ChIP-seq
☐	☒ Flow cytometry
☒	☐ MRI-based neuroimaging

Antibodies

Antibodies used	Flow cytometry: Human FcR Binding Inhibitor (1:5, eBiosciences, 16-9161-73), APC-Cy7 Mouse Anti-Human CD19 (1:20, BD Pharmingen, 348794), APC Mouse Anti-Human CD11b (1:5, BD Pharmingen, 550019).<br>ChIPmentation: Rabbit anti-CTCF (2 µg, Diagenode, C15410210-50), Rabbit anti-SMC1A (2 µg, Abcam, ab9262), Rabbit anti-MED26 (2 µg, Bethyl Laboratories, A302-370), Drosophila spike-in antibody (1 µg, Biozol, 61686).
Validation	Validation was performed by the manufacturer. The antibodies were purified using immunogen affinity and validated by immunoprecipitation, immunohistochemical analysis, and western blotting.<br>- Human FcR Binding Inhibitor (eBiosciences, 16-9161-73) validated for flow cytometry (https://www.thermofisher.com/antibody/product/Fc-Receptor-Binding-Inhibitor-Antibody-Polyclonal/16-9161-73).<br>- APC-Cy7 Mouse Anti-Human CD19 (BD Pharmingen, 348794) validated for flow cytometry (https://www.bdbiosciences.com/en-us/products/reagents/flow-cytometry-reagents/clinical-discovery-research/single-color-antibodies-ruo-gmp/apc-cy-7-mouse-anti-human-cd19.348794?tab=format_details).<br>- APC Mouse Anti-Human CD11b (BD Pharmingen, 550019) validated for flow cytometry (https://www.bdbiosciences.com/en-ca/products/reagents/flow-cytometry-reagents/research-reagents/single-color-antibodies-ruo/apc-mouse-anti-human-cd11b.550019?tab=format_details).<br>- Rabbit anti-CTCF (2 µg, Diagenode, C15410210-50), ChIP-grade, validated for Western Blot and ChIP-seq (https://www.diagenode.com/en/p/ctcf-polyclonal-antibody-classic-50-mg).<br>- Rabbit anti-SMC1A (2 µg, Abcam, ab9262) validated for Western Blot and Immunoprecipitation (https://www.abcam.com/en-us/products/primary-antibodies/smc1a-antibody-ab9262?srsltid=AfmBOopPzSVjxgsokl23cisdMQFvcEjvAh9wVoN9u2UgEWLZLY1slpnW#). Validated for ChIP in Dluhosova et al., 2014: https://doi.org/10.1371/journal.pone.0092635.<br>- Rabbit anti-MED26 (2 µg, Bethyl Laboratories, A302-370) validated for Western Blot and Immunoprecipitation (https://www.thermofisher.com/antibody/product/CRSP7-Antibody-Polyclonal/A302-370A).<br>- Drosophila spike-in antibody (1 µg, Biozol, 61686) validated by Active Motif for ChIP-seq spike-in normalisation (https://www.activemotif.com/catalog/1091/chip-normalization).

Eukaryotic cell lines

Policy information about cell lines and Sex and Gender in Research

Cell line source(s)	The wild type Human B-cell Precursor Leukemia Cell Line (BLaER1) was a gift from the laboratory of Patrick Cramer (MPI-NAT, Goettingen). This cell line was originally created by the laboratory of Thomas Graf (CGR, Barcelona) in Rapino et al., 2013, Cell Reports from the parental B cell precursor leukemia (RCH-ACV, ACC 548) cell line. The cell line is also available commercially from Sigma-Aldrich (SCC165). The BLaER1-CTCF-mAID cell line was a gift from Grégoire Stik (CRG, Barcelona), and was created from the parental BLaER1 cell line in Stik et al., 2020, Nature Genetics.
Authentication	The BLaER1 cells were authenticated using the KaryoStat+ assay (Thermo Fisher). The BLaER1-CTCF-mAID cell line was created from the authenticated BLaER1 line and not further authenticated.
Mycoplasma contamination	All cell lines tested negative for mycoplasma contamination.
Commonly misidentified lines<br>(See ICLAC register)	No commonly misidentified lines were used.

Plants

Seed stocks	n/a
Novel plant genotypes	n/a
Authentication	n/a

ChIP-seq

Data deposition

☒ Confirm that both raw and final processed data have been deposited in a public database such as GEO.

☒ Confirm that you have deposited or provided access to graph files (e.g. BED files) for the called peaks.

Data access links
May remain private before publication.

GEO link: https://www.ncbi.nlm.nih.gov/geo/query/acc.cgi?acc=GSE263641
Reviewer token: wxmfyuqqhrcptsh

Files in database submission

GSM8195732 BLaER, SMC1A_ChIP_0h_rep1
GSM8195733 BLaER, SMC1A_ChIP_0h_rep2
GSM8195734 BLaER, SMC1A_ChIP_0h_rep3
GSM8195735 BLaER, SMC1A_ChIP_12h_rep1
GSM8195736 BLaER, SMC1A_ChIP_12h_rep2
GSM8195737 BLaER, SMC1A_ChIP_12h_rep3
GSM8195738 BLaER, SMC1A_ChIP_24h_rep1
GSM8195739 BLaER, SMC1A_ChIP_24h_rep2
GSM8195740 BLaER, SMC1A_ChIP_24h_rep3
GSM8195741 BLaER, SMC1A_ChIP_72h_rep1
GSM8195742 BLaER, SMC1A_ChIP_72h_rep2
GSM8195743 BLaER, SMC1A_ChIP_72h_rep3
GSM8195744 BLaER, SMC1A_ChIP_96h_rep1
GSM8195745 BLaER, SMC1A_ChIP_96h_rep2
GSM8195746 BLaER, SMC1A_ChIP_96h_rep3
GSM8195747 BLaER, MED26_ChIP_0h_rep1
GSM8195748 BLaER, MED26_ChIP_0h_rep2
GSM8195749 BLaER, MED26_ChIP_0h_rep3
GSM8195750 BLaER, MED26_ChIP_12h_rep1
GSM8195751 BLaER, MED26_ChIP_12h_rep2
GSM8195752 BLaER, MED26_ChIP_12h_rep3
GSM8195753 BLaER, MED26_ChIP_24h_rep1
GSM8195754 BLaER, MED26_ChIP_24h_rep2
GSM8195755 BLaER, MED26_ChIP_24h_rep3
GSM8195762 BLaER subclone CTCF-AID, CTCF_ChIP _96h_control_rep1
GSM8195763 BLaER subclone CTCF-AID, CTCF_ChIP _96h_control_rep2
GSM8195764 BLaER subclone CTCF-AID, CTCF_ChIP _96h_depl_rep1
GSM8195765 BLaER subclone CTCF-AID, CTCF_ChIP _96h_depl_rep2
GSM8195756 BLaER, MED26_ChIP_72h_rep1
GSM8195757 BLaER, MED26_ChIP_72h_rep2
GSM8195758 BLaER, MED26_ChIP_72h_rep3
GSM8195759 BLaER, MED26_ChIP_96h_rep1
GSM8195760 BLaER, MED26_ChIP_96h_rep2
GSM8195761 BLaER, MED26_ChIP_96h_rep3
GSM8740797 BLaER subclone CTCF-AID, SMC1A_ChIP _96h_control_rep1
GSM8740798 BLaER subclone CTCF-AID, SMC1A_ChIP _96h_control_rep2
GSM8740799 BLaER subclone CTCF-AID, SMC1A_ChIP _96h_depl_rep1
GSM8740800 BLaER subclone CTCF-AID, SMC1A_ChIP _96h_depl_rep2
GSM8740801 BLaER subclone CTCF-AID, MED26_ChIP _96h_control_rep1
GSM8740802 BLaER subclone CTCF-AID,MED26_ChIP _96h_control_rep2
GSM8740803 BLaER subclone CTCF-AID, MED26_ChIP _96h_depl_rep1
GSM8740804 BLaER subclone CTCF-AID, MED26_ChIP _96h_depl_rep2

Genome browser session
(e.g. UCSC)

https://genome-euro.ucsc.edu/cgi-bin/hgTracks?db=hg38&lastVirtModeType=default&lastVirtModeExtraState=&virtModeType=default&virtMode=0&nonVirtPosition=&position=chr6%3A133824244%2D136599852&hgsid=348343346_rZSyfdVVteG8voU2PaaW4P2jFgAO

Methodology

Replicates	ChIPmentation experiments for MED26 and SMC1A were performed in 3 biological replicates per condition; ChIPmentation experiments for CTCF were performed in 2 biological replicates per condition; ChIPmentation experiments for MED26 and SMC1A after CTCF depletion were performed in 2 biological replicates per condition.
Sequencing depth	The samples were sequenced using the NextSeq550 Illumina platform (75-bp paired-end reads) to a sequencing depth of ~20 M reads per sample.
Antibodies	ChIPmentation: Rabbit anti-CTCF (2 µg, Diagenode, C15410210-50), Rabbit anti-SMC1A (2 µg, Abcam, ab9262), Rabbit anti-MED26 (2 µg, Bethyl Laboratories, A302-370), Drosophila spike-in antibody (1 µg, Biozol, 61686).
Peak calling parameters	Paired-end reads were processed for adapter removal and duplicate filtering and mapped to the hg38 reference genome using Bowtie2. Peak calling was performed with MACS2 (consensus peaks with parameter q = 0.1 were selected). All peak profiles were generated using Deeptools.
Data quality	The quality of the data was assessed by comparing CTCF-control samples to available CTCF ChIP-seq data (Stik, et al., 2020, Nature Genetics). As SMC1A and MED26 ChiPmentation experiments have been performed for the first time in this cell line, data quality was assessed by comparing 3 biological replicates.
Software	Paired-end reads were processed for adapter removal and duplicate filtering and mapped to the hg38 reference genome using Bowtie2. Peak calling was performed with MACS2. All peak profiles were generated using Deeptools.

Flow Cytometry

Plots

Confirm that:

☒ The axis labels state the marker and fluorochrome used (e.g. CD4-FITC).

☐ The axis scales are clearly visible. Include numbers along axes only for bottom left plot of group (a 'group' is an analysis of identical markers).

☒ All plots are contour plots with outliers or pseudocolor plots.

☒ A numerical value for number of cells or percentage (with statistics) is provided.

Methodology

Sample preparation	The BLaER1 cells were cultured and differentiated and flow cytometry analysis was performed at different differentiation stages for 2 biological replicates. Cells were harvested, washed with PBS, blocked with human Fc Receptor Binding Antibody, stained with antibodies, washed with PBS and analyzed. A more detailed protocol is described in the Methods section.
Instrument	Sony SH800 Cell Sorter (MPI-NAT, Goettingen).
Software	FloJo v.10.8.1
Cell population abundance	Cells were analyzed for the expression of the cell surface markers CD19 (B cell marker) and CD11b (iMacs marker). All the BLaER1 cells were positive for CD19 and after 7 days of transdifferentiation around 90 % of the cells lost the CD19 marker and acquired the CD11b marker.
Gating strategy	Initial gating was performed based on forward and side scatters to identify single cells and exclude cell debris. Firstly, a gate was set on forward scatter height (FSC-H) vs forward scatter area (FSC-A) plot to exclude doublets. Secondly, a gate was set on side scatter height (SSC-H) vs forward scatter area (FSC-A) plot to exclude debris cells. Thirdly, a gate was set on the histogram to include only GFP positive, alive cells. Quadrant gates were based on non-differentiated B-cell population as negative control. They were set on 0 h sample, which is CD19 positive and CD11b negative. All relevant data are shown in Extended Data Fig. 1b.

☒ Tick this box to confirm that a figure exemplifying the gating strategy is provided in the Supplementary Information.
